# Stimulation of Let-7 Maturation by Metformin Improved the Response to Tyrosine Kinase Inhibitor Therapy in an m6A Dependent Manner

**DOI:** 10.3389/fonc.2021.731561

**Published:** 2022-01-06

**Authors:** Kai Li, Shan Gao, Lei Ma, Ye Sun, Zi-Yang Peng, Jie Wu, Ning Du, Hong Ren, Shou-Ching Tang, Xin Sun

**Affiliations:** ^1^ Department of Thoracic Surgery, Department of Thoracic Surgery and Oncology, Cancer Center, The First Affiliated Hospital of Xi’an Jiaotong University, Xi’an City, China; ^2^ Department of Anesthesiology and Perioperative Medicine, Operating Centre, The First Affiliated Hospital of Xi’an Jiaotong University, Xi’an City, China; ^3^ Department of Anesthesiology and Operation, Operating Centre, The First Affiliated Hospital of Xi’an Jiaotong University, Xi’an City, China; ^4^ University of Mississippi Medical Center, Cancer Center and Research Institute, University of Mississippi, Jackson, MS, United States

**Keywords:** therapy resistance, cancer stem-like cells, tyrosine kinase inhibitor, n6-methyladenosine, miRNAs maturation

## Abstract

The molecular mechanism of the tyrosine kinase inhibitor (TKI) resistant lung adenocarcinoma is currently unclear, and the role of methylated adenosine at the N6 position in the resistance of cancer stem cells (CSCs) therapy is unknown. This study identified a novel and effective strategy to enhance TKIs therapy response. We first confirmed the sensitization of Metformin enforcing on Osimertinib treatment and revealed the mature miRNAs signatures of the Osimertinib resistant H1975 and HCC827 cells. Let-7b expression was stimulated when adding Metformin and then increasing the therapy sensitivity by decreasing the stem cell groups expanding. Methyltransferase-like 3 (METTL3) increased the pri-Let-7b, decreased both the pre-Let-7b and mature Let-7b, attenuating the Let-7b controlling of stem cell renewal. The addition of Metformin increased the bindings of DNA methyltransferase-3a/b (DNMT3a/b) to the METTL3 promoter. With the help of the readers of NKAP and HNRNPA2B1, the cluster mediated m6A formation on pri-Let-7b processing increased the mature Let-7b, the key player in suppressing Notch signaling and re-captivating Osimertinib treatment. We revealed that the maturation processing signaling stimulated the methylation regulation of the miRNAs, and may determine the stemness control of the therapy resistance. Our findings may open up future drug development, targeting this pathway for lung cancer patients.

## Introduction

Lung cancer is the leading cause of cancer-related death worldwide (1, 2), and early detection, early diagnosis, and early treatment are key to its treatment (3, 4). Many steps are taken in China for lung cancer prevention (5–7). Therapy strategies against NSCLC could be classified as chemotherapy, radiotherapy, targeted therapy, and immunotherapy, and targeting therapy of EGFR-tyrosine kinase inhibitors (TKIs) is continuously gaining prominence in research (8). However, patients can encounter resistance, and therapy eventually fails (9, 10). The TKIs have been applied in clinical treatments for years with successful delivery strategies. One commonly encountered problem is the emergence of resistance to the first generation of TKIs due to the acquired EGFR mutation (exon 18, 19, 21) and L858R/T790M point mutation (10–12). This can be managed by the third generation EGFR inhibitors such as Osimertinib, which effectively prolong progress free survival (PFS) and the overall survival (OS) of advanced or resistant NSCLC patients. The ongoing studies will determine whether the Osimertinib can be used as first line treatment, for now, the drug is also believed to be active in NSCLC patients with brain metastasis (13). However, there is no drug available currently to treat or delay resistance to Osimertinib.

The lack of effective treatment for Osimertinib resistant tumors was due to a poor understanding of the factors contributing to therapy response. Cancer stem-like cells (CSCs), also known as cancer initiating cells, function to generate the malignancy group and contribute to therapy resistance (10–12). In recent years, our focus has been on the non-coding RNAs controlling the stem cell renewal, and we observed that mature miRNAs determined stem cell fate by many mechanisms (13). In this study, we tentatively explored the latent roles of miRNAs in Osimertinib treatment response and tried to explore methylation related miRNA maturation in the generation of resistance. We discovered that Metformin can regulate the self-renewal of lung cancer stem cells and their malignant phenotypes. Our observation will pave the way for future drug development using metformin to address the resistance to EGFR TKIs and may lead to better lung cancer therapy in both metastatic and adjuvant settings.

## Methods

### Cells culturing and Osimertinib Resistant Cells Establishment

The lung adenocarcinoma cell lines of H1975 and HCC827 were obtained from the American Type Culture Collection (ATCC, Manassas, VA, USA), and cultured in RPMI-1640 medium supplemented with 10% fetal bovine serum (FBS), 100 μg/mL penicillin, and 100 μg/mL streptomycin. Cells were maintained in an incubator with 5% CO_2_ at 37°. We established the Osimertinib resistant H1975 cells (H1975OR) and the resistant HCC827 cells (HCC827OR) by gradually increasing concentrations of Osimertinib for 72 hours with a recovery period between treatments, as described in detail in the results section.

### RNA Microarray, qRT-PCR, and Data Analysis

Total RNAs were extracted by using Trizol reagent (Invitrogen, CA, USA), according to the manufacturer’s instructions, and the arrays were scanned to images using the Agilent Scanner G2505C. Data were normalized and processed using the R software Limma-package. The differentially expressed miRNAs were screened through fold change>2. For qRT-PCR, the total extracted RNAs were reverse transcribed using a PrimeScript RT reagent Kit with gDNA Eraser (TAKARA, Dalian, China), and the complementary DNA templates were amplified by using SYBR Premix Ex Taq (TAKARA), as previously reported (14, 15). Kyoto Encyclopedia of Genes and Genomes (KEGG) pathway analysis was conducted to evaluate the attributes of miRNAs in pathways (https://www.genome.jp/kegg/).

### Stem-Like Cell Isolation and Functional Identification

The ALDH1A1 phenotype is proven to be one of the most indicative markers referring to the stem cells group. Specifically, ALDEFLUOR is supplied in the form of Bodipy-aminoacetalde-hyde Diethyl-Acetal, which by itself is not a substrate of ALDH, and briefly, A) ALDEFLUOR ACTIVATION, B) CELL SAMPLE PREPARATION, C) ALDEFLUOR ASSAY, D) FLOW-C-YTOMETER SET-UP AND DATA ACQUISITION, according to the manufacturing information of ALDEFLUOR Kit (Stem-cell Technology, Canada). Stem cell enrichment was achieved by spheres culturing in suspension cultivation with a special made medium as previously proven in our earlier study (14, 16).

### RNA Immunoprecipitation and Chromatin Immunoprecipitation Assays

Antibodies against EGFR (1:3000, EP38Y, ab52894, Rabbit monoclonal, Abcam), p-EGFR (Tyr-845 phosphorylated EGFR, 1:1000, 12A3, sc-57542, mouse monoclonal, Santa Cruz), Notch1 (1:3000, ab65297, Rabbit polyclonal, Abcam), Notch2 (1:3000, ab137665, Rabbit polyclonal, Abcam), NCSTN (1:1000, 14071-1-AP, Nicastrin Polyclonal antibody, Protein-tech), Vinculin (1:5000, #4650, Rabbit polyclonal, Cell Signaling), OCT-4 (1:5000, #2750S, Rabbit polyclonal, Cell Signaling), Snai1 (1:1000, 13099-1-AP, Rabbit polyclonal, Thermo-Fisher Scientific), Klf-4 (1:1000, 11880-1-AP, KLF4 Polyclonal antibody, Rabbit polyclonal, Protein-tech), WTAP (1:500, D-7, sc-374280, mouse monoclonal, Santa Cruz), METTL3 (1:3000, 15073-1-AP, METTL3 Polyclonal antibody, Rabbit polyclonal, Protein-tech), METTL14 (1:2000, 26158-1-AP, METTL14 Polyclonal antibody, Rabbit polyclonal, Protein-tech), NKAP (1:3000, ab229125, C-Terminal, Rabbit polyclonal, Abcam), HNRNPA2B1 (1:5000, DP3B3, ab6102, Mouse monoclonal, Abcam), DGCR8 (1:3000, PA5-78510, Rabbit polyclonal, Invitrogen, Thermo-Fisher), DROSHA (1:1000, ab58589, Goat Polyclonal, Abcam), were used in the study and were described in detail for the following application.

RNA immunoprecipitation assays (RIP) were performed using the Magna RIP RNA-Binding Protein Immuno-precipitation Kit (17-700, Millipore, Sigma-Aldrich). Total RNA and isotype control for each antibody was assayed as input control and IgG respectively, and simultaneously, the co-precipitated RNAs were detected by qRT-PCR. To detect the m6A level in Let-7b, total RNAs were subjected to ribosome RNA depletion using the Ribo-Zero Gold rRNA Removal Kit (Illumina) first, and Ribo-off RNAs were subjected to RNA Fragmentation Reagents (AM8740, Ambion, Applied Biosystem). Precipitation was performed using anti-m6A antibody (1:1000, #202003, Rabbit polyclonal, Synaptic Systems GmbH, SYSY) previously bound to magnetic Dynabeads using a Co-Immunoprecipitation Kit (14321D, Thermo-Fisher), and then incubated with fragmented RNAs.

For western blot analysis, the proteins from cell extracts were separated by 10% SDS-PAGE electrophoresis and cut prior to hybridization. After being transferred onto PVDF membranes, the protein adherent membranes were then incubated with specific antibodies. ECL Blotting Detection Reagents (Merck Millipore) were used for final blot development.

Chromatin immunoprecipitation (ChIP) assays were performed using the kit (#17-295, Sigma-Aldrich, MERCK). Cells were cross-linked with 1% formaldehyde, lysed, and sonicated on ice to generate DNA fragments, pre-cleared DNA of each sample was saved as input fraction and then used for immunoprecipitation with specific antibodies, as were listed above. IgG was included as a nonspecific control.

### DNA Methylation Analysis

DNA from different groups was extracted using the TIANamp Genomic DNA Kit (DP130227, #DP304, TIANGEN), and purified DNA samples were subsequently subject to bisulfite conversion using the EpiTect Fast Bisulfite Conversion Kits (#59826, TIANGEN). Methylation-specific PCR was performed using EpiTect Methy-Light PCR Kits (#59436, TIANGEN) with specific primers targeting the CpG island in the METTL3 promoter.

### Statistical Analysis

The relative expression of RNA was calculated using the 2^-ΔΔCt^ method, where Ct represents the threshold cycle. Graph-Pad Prism 6.0 was used for data analysis and for plotting. Wilcoxon matched pairs tests were used to compare the circRNA expression levels of two groups. p<0.05 was considered statistically significant.

## Results

### Alterations in Biology and Proliferation Patterns of the Osimertinib Resistant Lung Cancer Adenocarcinoma Cells

The drug sensitivity was checked and analyzed using data from Genomics of Drug Sensitivity in Cancer at the SANGER site (https://www.cancerrxgene.org/). H1975 cells were originally sensitive to Osimertinib treatment (**Supplemental Figure 1**), and all related therapeutic agents were listed with a specific score, as listed in **Supplemental Figure 1B**. The IC_50_ value of Osimertinib for H1975 was calculated approximately at 60 nM (**Supplemental Figure 1C**), while the IC_50_ value of H1975OR was 2μM (**Supplemental Figure 1C**). The signatures of resistant H1975OR cells were identified with activated Notch signaling and mutant KRAS status (**Supplemental Figures 1D, E**), which was applied for correlation analysis in detail (**Supplemental Figure 1F**).

The established Osimertinib resistant adenocarcinoma cells of H1975OR were analyzed for gene-type signatures, as was illustrated in **Supplemental Figure 2**. The overall view of the differentially expressed non-coding RNAs was embedded in the heat map (**Supplemental Figure 3A**), and the dysregulated miRNAs were chosen for their significant abnormalities (GSE184980). The box plot showed the distribution of RNA intensities in the six samples referring to quality control (**Supplemental Figure 3B**), and the distribution of normalized intensities was almost the same for the miRNAs (**Supplemental Figure 3C**). The horizontal comparisons were made referring to the irregulated miRNAs. Given that only the miRNAs exhibited the most significant differences, the selected miRNAs were double-checked with qPCR detection (**Supplemental Figure 3D**), of which, hsa-Let-7b and hsa-Let-7d were suppressed greatly in Osimertinib resistant H1975OR cells.

A Volcano plot exhibited the differences in miRNA expression as marked with red and blue tags (**Supplemental Figure 3E**). The meaningful prediction of the GO terms and pathways were chosen and ranked according to enrichment scores, and the most significantly enriched biological process terms were regulative transcription and small molecular metabolic processes (**Supplemental Figure 3F**, Left). The most significantly enriched cellular component terms are located in the nucleus and cytoplasm (**Supplemental Figure 3F**, middle). The most significantly enriched molecular function terms were protein binding (**Supplemental Figure 3F**, right). GO analysis was applied for predicting and analyzing the possible functional pathways in which altered miRNAs may participate, and Let-7b was set as the potential candidate for future studies.

### Osimertinib Resistant Lung Adenocarcinoma Cells Harbored Decreased Let-7 Family of miRNAs and Notch Signaling

**Figure 1:**
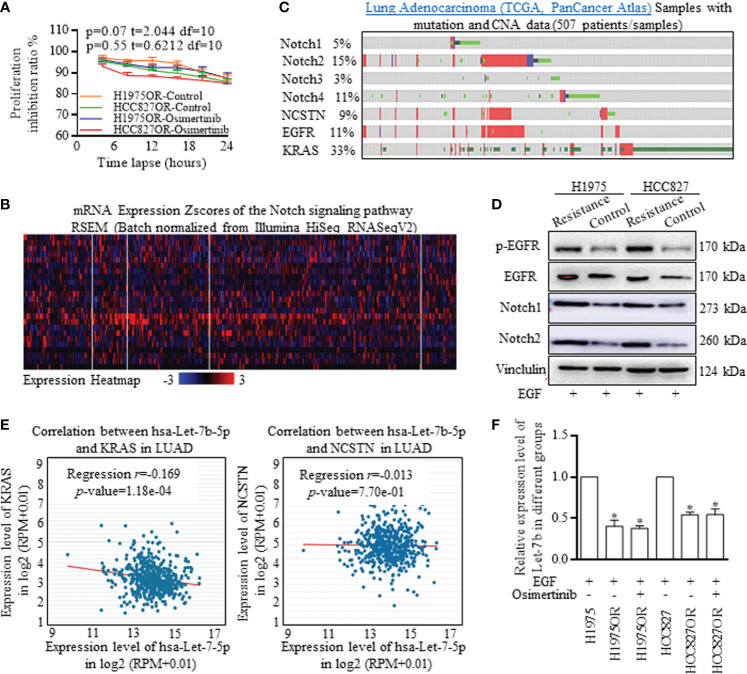
Notch signaling signatures and Let-7b expression deviations in Osimertinib resistant cells. **(A)** Proliferation inhibitive ratios were detected for defining the Osimertinib resistant H1975OR cells and HCC827OR cells. **(B)** The samples of lung Adenocarcinoma from the TCGA database (Pan-Cancer Atlas) were analyzed with mutation and CNA data, and Notch signaling participants were universally activated. **(C, D)** Key functional factors of Notch signaling were primarily screened and confirmed by western blotting, and Notch signaling factors were overexpressed in resistant cells (The grouping of gels/blots were cropped from different parts, and the full-length gels could be referred to in the supplemental data). **(E)** The inverse relationship between Let-7b and key Notch signaling activators was defining with using data from Star-Base Project. **(F)** Let-7b decreased significantly in Osimertinib resistant H1975OR and HCC827OR cells (**Figure 2F**).

The H1975 cell line bearing T790M/L858R mutations was selected for its resistance to first generation TKIs, and the HCC827 cell line bearing sensitive EGFR mutations was selected for another test group. We successfully established the Osimertinib resistant H1975OR cells and HCC827OR cells (**Figure 1A**). The samples of lung Adenocarcinoma from the TCGA database (*Pan-Cancer Atlas/Firehose Legacy*) were analyzed with mutation and CNA data. Notch signaling participants were universally activated, and the detailed expression patterns were illustrated in a heat map (**Figure 1B**). Notch signaling activation correlated with the stem cells’ renewal and therapy resistance, thus, we then explored the co-activation of EGFR signaling and Notch signaling. Key functional factors of Notch signaling were primarily screened (**Figure 1C**), which were correlated with poorer survival expectance (**Supplemental Figure 4A**), and the results were confirmed by western blotting in resistant lung cancer cells (**Figure 1D** and **Supplemental Figure 5**). We also detected the inverse relationship between Let-7b and key Notch signaling activators (**Figure 1E**, *Data Source: StarBase v3.0*), Let-7b, alone, indicated better survival expectance in lung adenocarcinoma, but failed in lung squamous cancer (**Supplemental Figures 4B, C**). Let-7b decreased significantly in Osimertinib resistant H1975OR and HCC827OR cells (**Figure 1F**), strongly suggesting the inverse interaction between Let-7b and Notch signaling associated treatment resistance.

### Resistant Lung Adenocarcinoma Consists of More Stem-Like Cells

**Figure 2:**
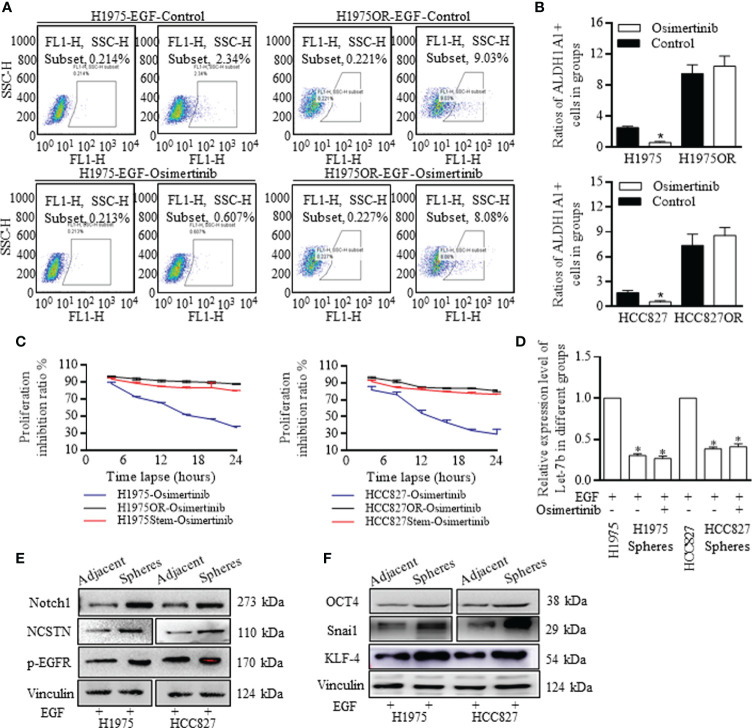
Notch signaling activation dependent stem cells renewal was responsible for Osimertinib resistance. **(A, B)** The ratio of ALDH1A1 (ALDH, ALDH1) positive cells accounted for less than 3% in H1975 and HCC827 cells, and Osimertinib decreased the ALDH1 positive stem cells ratio of H1975 and HCC827 effectively, however in H1975OR and HCC827OR cells, the ratios increased incredibly, and the stem cells ratio did not react to Osimertinib treatment. **(C)** Proliferation inhibition ratio was essentially the same between H1975 cells and H1975OR cells, between HCC827 cells and HCC827OR cells. **(D)** Let-7b decreased significantly in the spheres of H1975 cells and HCC827 cells, and Osimertinib did not change the Let-7b expression compared to the negative control. **(E, F)** Stem cells were identified with overexpressed stem associated markers, and the key notch signaling actors were excessively activated in spheres of stem cells (The grouping of gels/blots were cropped from different parts, and the full-length gels could be referred to in the **Supplemental Data**). *P < 0.05

The phenotype of ALDH1A1 positive cells was identified and treated as the stem cells group, with the ability of higher self-renewal, determining the poorer survival expectance. Resistant H1975OR cells and HCC827OR cells consisted of more ALDH1A1+ cells than primary cells (**Figures 2A, B**), which could form many more spheres than the adjacent cells (data not shown). Moreover, the ALDH1A1 cells group was naturally resistant to Osimertinib, and the proliferative differences were not significant when receiving Osimertinib (**Figure 2C**). Similarly, Let-7b decreased significantly in stem cells of spheres (**Figure 2D**), and we confirmed that the stem cells group was responsible for therapy resistance, and therefore detected the Notch signaling characteristics in spheres of H1975 and HCC827 stem cells (**Figure 2E** and **Supplemental Figure 6**), and the functional levels of EGFR and Notch signaling activators were much higher in the stem cells of spheres, which harbored stemness signatures (**Figure 2F** and **Supplemental Figure 7**).

### Metformin Sensitized the Osimertinib Resistant Cells by Decreasing the Stem Cells Ratio

**Figure 3:**
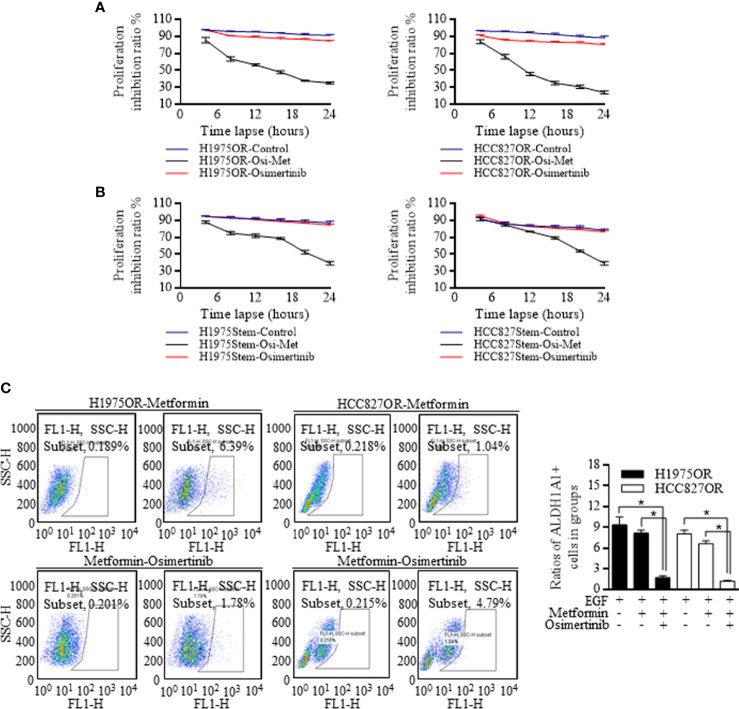
Metformin decreased the stem cells ratio and sensitized the resistant lung cancer cells to Osimertinib. **(A)** The proliferation ratio of H1975OR cells and HCC827OR cells decreased more greatly than the control groups when culturing with Metformin. **(B)** The proliferative index of cancer cells from the spheres of H1975 and HCC827 cells decreased significantly when co-cultured with Metformin. **(C)** Metformin decreased the ratios of ALDH1A1 positive cells in the group receiving Osimertinib treatment, however, the stem cells ratio did not change greatly when using Metformin alone. *P < 0.05.

The H1975OR cells and HCC827OR cells responded better to Osimertinib than the control groups when culturing with 5 mmol of Metformin (**Figure 3A**). Moreover, when the sphere cells of H1975 and HCC827 were co-cultured with Metformin, the proliferative index also decreased (**Figure 3B**). To further locate the implicated mechanisms, we found that Metformin decreased the ratios of ALDH1A1 cells in the group receiving Osimertinib, indicating that stem cells accounted for therapy resistance, which could be attenuated by Metformin (**Figure 3C**). Moreover, we noticed that the stem cell ratio did not change as significantly as the combination group when using Metformin alone, indicating its assistant role, but not dominant role in facilitating therapy response.

### Metformin Sensitization of Osimertinib Resistant Cells Was Dependent on Mature Let-7b Overexpression

**Figure 4:**
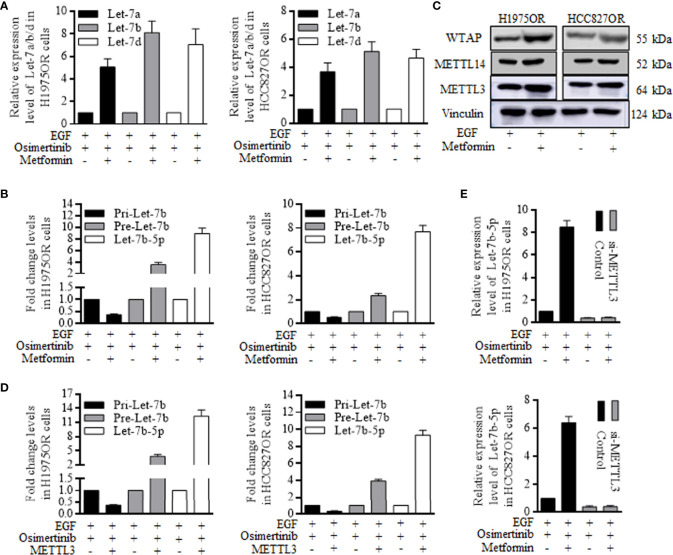
Metformin increased the mature Let-7b through stimulating METTL3 expression and re-sensitized the resistant lung cancer cells. **(A)** Let-7b increased significantly in the resistant cells group when receiving Metformin and Osimertinib. **(B)** Metformin caused the inconsistent levels of pri-let-7b and pre-let-7b, stimulating the miRNA maturation. **(C)** METTL3, METTL14, and Wilms tumor 1-associated protein (WTAP) are three components of the writer protein complex in m6A process, and Metformin only increased the METTL3 expression greatly. **(D)** METTL3 alone remarkably decreased pri-Let-7b, and increased both pre-Let-7b and Let-7b-5p (the grouping of gels/blots were cropped from different parts, and the full-length gels could be referred to in the supplemental data). Knocking down of METTL3 abolished the Metformin effects of induction of Let-7b-5p overexpression in H1975OR cells (**E**, above) and HCC827OR cells (**E**, below).

The let-7 family of Let-7a, Let-7b, and Let-7d were detected when receiving combined Metformin and Osimertinib, and Let-7b increased significantly in the resistant cells group (**Figure 4A**). The functional miRNAs of mature patterns were processed by a nuclear microprocessor complex composed of DGCR8/DROSHA (17, 18), and were then exported to the cytoplasm as RNA induced silencing complex (RISC) to perform certain functions. We found inconsistent levels of pri-let-7b and pre-let-7b, indicating that Metformin functioned through perturbing the let-7b maturation (**Figure 4B**). Since methyltransferase plays a key role in microRNA maturation by catalyzing m6A formation (19–23), we examined expression levels of Methyltransferase-like 3 (METTL3), Methyltransferase-like 14 (METTL14), and Wilms tumor 1-associated protein (WTAP), three components of the writer protein complex in m6A process. Metformin increased the METTL3 expression significantly (**Figure 4C** and **Supplemental Figure 8**), and the METTL3 alone remarkably decreased pri-Let-7b and increased both pre-Let-7b and Let-7b-5p (**Figure 4D**). Similarly, knocking down METTL3 abolished the Metformin effects of induction of Let-7b-5p overexpression in H1975OR cells (**Figure 4E**, above) and HCC827OR cells (**Figure 4E**, below).

### Metformin Promotes the METTL3 Mediated Let-7b Maturation by Suppressing the Promoter Methylation Level

**Figure 5:**
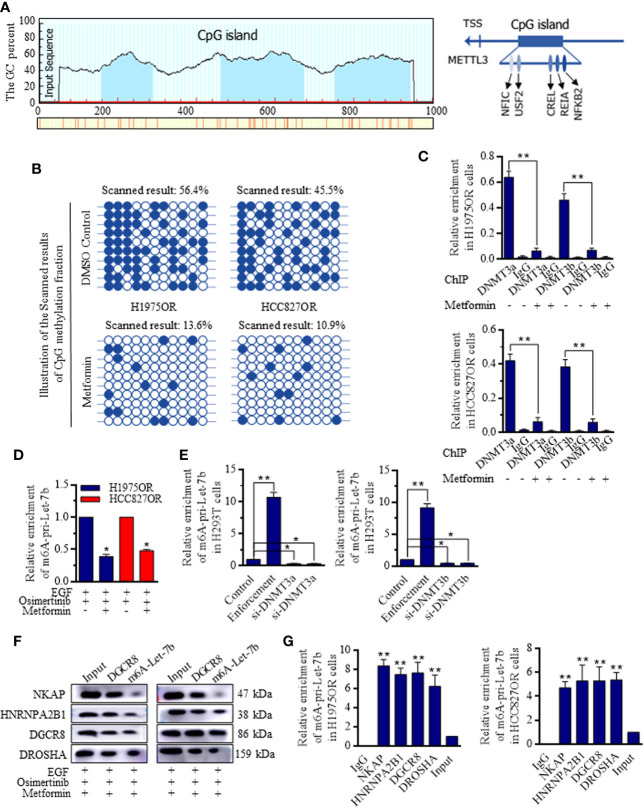
Metformin inhibited DNMT3 promoter activity and facilitated the METTL3 mediated Let-7b maturation. **(A)** The possible CpG island upstream of the METTL3 locus referring to Let-7b was generated and exhibited. **(B)** Cells treated with Metformin had significantly lower methylation within METTL3 CpG island in comparison to cells without the exposure. **(C)** Metformin effectively reduced the bindings of DNMT3a and DNMT3b to the METTL3 promoter. **(D)** m6A-specific RNA immunoprecipitation (RIP) coupled qRT-PCR analysis referring to METTL3-mediated m6A on the pri-Let-7b maturation revealed that m6A-pri-Let-7b decreased significantly when the Osimertinib resistant cells received the Metformin. **(E)** Overexpressing either DNMT3a or DNMT3b into the H293T cells could reduce the m6A-pri-Let-7b levels, and knocking down METTL3 stimulated the m6A-pri-Let-7b expression. **(F)** RIP-coupled RT-PCR and western blotting in m6A-Let-7b and DGCR8 groups under Metformin treatment showed that both NKAP and HNRNPA2B1 bound with DGCR8 and m6A-Let-7b significantly, and m6A-Let-7b existed with DGCR8 and DROSHA to perform maturation functions (The grouping of gels/blots were cropped from different parts, and the full-length gels could be referred to in the **Supplemental Data**). RIP-coupled qRT-PCR indicated the bindings between NKAP/HNRNPA2B1 and m6A-Let-7b in H1975OR (**G**, left) cells and HCC827OR cell (**G**, right).

JASPAR analysis indicated the possible CpG islands upstream of the METTL3 locus (**Figure 5A**, http://www.urogene.org/methprimer/), and the cells treated with Metformin had significantly lower methylation within this CpG island compared to cells without the exposure (**Figure 5B**). Quantitative chromatin immunoprecipitation (ChIP) assays showed that Metformin effectively reduced the bindings of DNA methyltransferase (DNMT)-3a and DNMT-3b to the METTL3 promoter (**Figure 5C**). To determine how the METTL3 activates the Let-7b maturation, m6A-specific RNA immunoprecipitation (RIP) coupled qRT-PCR analysis was performed, and the effects of METTL3-mediated m6A on the pri-Let-7b maturation revealed that m6A-pri-Let-7b decreased greatly when the Osimertinib resistant cells received the Metformin (**Figure 5D**). Further, we identified that enforcing either DNMT3a or DNMT3b into the H293T cells could reduce the m6A-pri-Let-7b levels (**Figure 5E**). NKAP and HNRNPA2B1 were both defined as crucial m6A readers to perform the final functional process (24–26), and results showed that both NKAP and HNRNPA2B1 bound with m6A-Let-7b significantly, and m6A-Let-7b existed with DGCR8 and DROSHA to perform maturation functions (**Figure 5F** and **Supplemental Figure 9**). Similarly, RIP-coupled qRT-PCR indicated the bindings between NKAP/HNRNPA2B1 and m6A-Let-7b in H1975OR (**Figure 5G**, left) cells and HCC827OR cells (**Figure 5G**, right).

### Increased Let-7b Was Critical for Re-Sensitizing the Osimertinib Treatment and Was Dependent on Inactivating Notch Signaling

**Figure 6:**
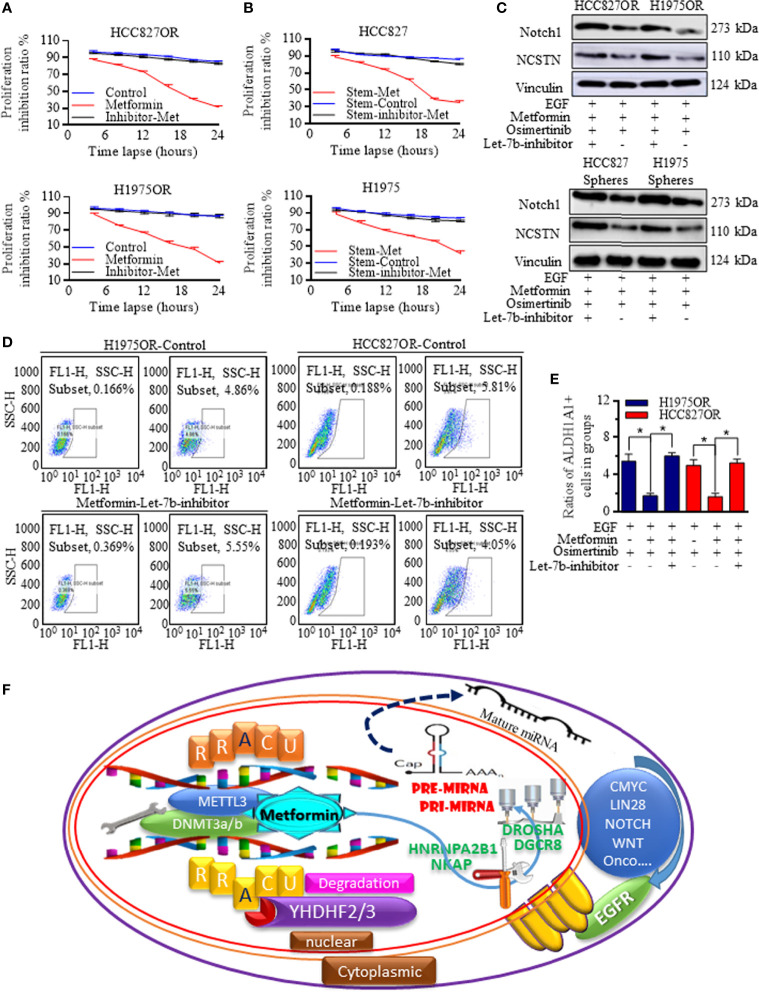
Let-7b was critical for sensitizing the Osimertinib treatment. Let-7b inhibition greatly abolished the Metformin effects in resistant cells **(A)** and in stem cells **(B)** when receiving Osimertinib, with Notch signaling re-activation (**C**, the grouping of gels/blots were cropped from different parts, and the full-length gels could be referred to in the **Supplemental Data**). **(D, E)** Let-7b inhibition specifically diminished the Metformin decreasing of stem cells group. **(F)** The regulative connections and the mechanistic m6A regulations of Let-7 were generated and drawn by PowerPoint and were illustrated as one image. *P < 0.05.

To determine the critical role of Let-7b in Metformin induction of Osimertinib sensitization, Let-7b expression was manipulated, and inhibitors referring to Let-7b greatly abolished the Metformin effects in resistant cells (**Figure 6A**) and stem cells (**Figure 6B**) when receiving Osimertinib, with Notch signaling re-activation (**Figure 6C** and **Supplemental Figure 10**). Similarly, Let-7b inhibition specifically diminished the Metformin decreasing of stem cells group (**Figures 6D, E**), and these results proved the dominant role of Let-7b in Metformin affected functional signaling. The regulative connections and the mechanistic m6A regulation of Let-7 were illustrated as a scheme (**Figure 6F**).

## Discussion

Therapeutics resistance has always been an issue when fighting against cancer, and a large number of studies have attempted to search for drug resistance-related genes (27, 28). Generally, chemotherapy and radiotherapy perished most cancer cells in cycles of treatments, however, steady cells with lower proliferation ability successfully survived. These silent cells did not react to agents admiring the rapidly proliferating cells. The cancer group consisted of heterogeneous sub-clones, and the cancer stem cells, also known as initiating cells and stem-like cells, were related to cancer initiation, progression, resistance, and recurrence. However, the reaction of stem cells and the changes in their gene signatures to targeted therapy with TKIs has never been explored.

TKIs targeting EGFR are used to treat lung cancer patients with EGFR amplification and mutations. They have significantly improved the survival of the treated patient population. They also improved the quality of life for these patients. However, most patients will inevitably develop the disease progression and TKI resistance (29, 30). Usually, when the L858R/T790M mutation emerges, the third generation such as Osimertinib can be used to prolong the survival time and control the disease progression. However, there is no strategy or TKIs available to treat or overcome Osimertinib resistance. We studied the non-coding RNAs’ profile and stem cells’ characteristics to reveal the latent mechanisms relating to Osimertinib resistance. Panels referring to non-coding RNAs were all analyzed, and several candidates with aberrant expressions were detected. Further, to explore the contribution of cancer stem cells to Osimertinib therapy resistance, we checked the proliferation ratio and re-confirmed the candidate miRNAs levels in the stem cells group. The ALDH1A1 cells exhibited similar miRNAs characteristics, and were originally resistant to Osimertinib, suggesting that the stem cells group may be the core force of group resistance. We further identified that the Notch signaling was activated in lung adenocarcinoma in the resistant tumors. Notch signaling is critical for maintaining proliferation and EMT-related stem cells’ initiation.

Metformin was reported to sensitize the TKIs’ effects, but its roles were unclear with unidentified mechanisms, and its facilitation referring to Osimertinib has not been explored. We first confirmed the sensitization of Metformin enforcing on Osimertinib and primarily revealed the mature miRNAs signatures of the Osimertinib resistant H1975 and HCC827 cells. Methylation was one of the RNA modifications patterns, among which, N6-methyladenosine (m6A) was mostly explored for its abundance in messenger RNA (mRNA) and non-coding RNAs. m6A modification, methylated adenosine at the N6 position (31, 32) is a dynamic and reversible process caused by cellular transformation, environmental hypoxia, or mutations accumulation (33–35), and regulates RNA transcription, processing, splicing, degradation, and translation (36, 37). However, its roles in non-coding RNAs were not clear. Functional m6A was accomplished with the assistance of writers, erasers, and readers, and in this study, we found the Osimertinib resistant lung cancer cells could be re-sensitized when adding Metformin, and resistant cancer stem cells were induced to renewal inhibition. Let-7b was selected as one representative inhibitor and could be stimulated when adding Metformin, contributing to Notch signaling inactivation. m6A writers included the methyltransferase complex, of which, the METTL3 could affect the miRNAs maturation by catalyzing the m6A formation in RNAs. Metformin increased the main members of the m6A writers. Among these, METTL3 decreased the pri-Let-7b and increased both the pre-Let-7b and mature Let-7b, which was illustrated in the schematic figure.

In conclusion, we identified one novel and effective strategy to enhance the TKIs’ therapy response, and the participation of Metformin decreased the bindings of DNMT3a/b to the METTL3 promoter with the help of the readers of NKAP and HNRNPA2B1. Together, the mediation of m6A formation on pri-Let-7b processing increased the mature Let-7b, whose key role is to suppress the Notch signaling and to re-captivate the Osimertinib treatment. Future *in vivo* studies will help to confirm the beneficial effects of Metformin usage prior to clinical trials. We revealed the maturation processing signaling filled up the methylation regulation of the miRNAs, which may dominate in alleviating the therapy resistance, and bring the prospective future for lung cancer treatments.

## Data Availability Statement

The data that support the findings of this study are available from the corresponding author upon reasonable request. The established Osimertinib resistant adenocarcinoma cells of H1975OR were analyzed for gene-type signatures, which could be viewed at https://www.ncbi.nlm.nih.gov/geo/query/acc.cgi?acc=GSE184980.

## Author Contributions

KL: Bioscientific experiments, Cells culturing. SG: Experimental tests, RNA/Protein tests. LM: Bioscientific experiments, Study designation. Z-YP: Experimental tests, Cells culturing. YS: Bioscientific experiments, Study designation. ND: RNA/Protein tests, Figures preparation. JW: Paper drafting, RNA/Protein tests, Statistical analysis, Database screening, Figures preparation. HR: Study designation, Statistical analysis. ST: Paper drafting, Study designation, Statistical analysis, Images quality control. XS: Paper drafting, Study designation, Statistical analysis, Database screening, Figures preparation, References cross checking. All authors contributed to the article and approved the submitted version.

## Funding

This experiment was supported by the National Science Foundation for Young Scientists of China, grant No. 81602597 (Referred to Xin Sun), National Science Foundation for Young Scientists of China, Grant No.82003140 (Referred to Guo-Dong Xiao). Foundation Research Project of Shaanxi Province, 2021SF-117 (Referred to XS). The Natural Science Basic Research Program of Shaanxi, No. 2018JM7017 (Referred to XS).

## Conflict of Interest

The authors declare that the research was conducted in the absence of any commercial or financial relationships that could be construed as a potential conflict of interest.

## Publisher’s Note

All claims expressed in this article are solely those of the authors and do not necessarily represent those of their affiliated organizations, or those of the publisher, the editors and the reviewers. Any product that may be evaluated in this article, or claim that may be made by its manufacturer, is not guaranteed or endorsed by the publisher.

## Supplementary Material

Supplementary Figure 1 RNA-panel analysis with gene ontology description and functional signaling predication. **(A)** More than 5000 non-coding RNAs were differentially expressed between the H1975 and H1975OR cells, and the representative heat map results were listed for illustrating the miRNAs, lncRNAs, circRNAs differences. **(B)** The distribution of RNA intensity in H1975 cells and H1975OR cells was confirmed. **(C)** Samples were detected triplicate for person correlation referring to quality control. **(D)** The abnormally expressed miRNAs were rechecked by qRT-PCR, and the expression variations of miRNAs were compared between qRT-PCR and microarray analysis results. **(E)** The volcano plot exhibited statistically significant differences in miRNAs expression. **(F)** Functional predication indicated the location and the pathways that alternative miRNAs may contribute to in the cellular biology and cancer initiation. **(G)** Signaling pathways that altered miRNAs may interact with were carried out with informatic GO analysis.

Supplementary Figure 2 The sensitivity of Osimertinib in treating Lung adenocarcinoma harboring mutant EGFR. The sensitivity analysis was carried out using shared data of Genomics of Drug Sensitivity in Cancer at the SANGER site. The Osimertinib sensitivity referring to lung cancer samples of PAN data was drafted with Z-score **(A)** and was rankly exhibited in table **(B)**. **(C)** H1975 cells were sensitive to Osimertinib with concentration much lower than IC_50_. **(D)** EGFR mutant lung cancer cells are very sensitive to Osimertinib treatment, comparing to that of lung cancer cells with wild type EGFR. **(E)** Adenocarcinoma cells with Kas-Mutant status were resistant to Osimertinib, exhibiting much higher IC_50_ score. **(F)** Most of the Notch signaling associated factors were primarily identified with resistance.

Supplementary Figure 3 Establishing the Osimertinib resistant cells. **(A)** Osimertinib was dissolved in dimethyl sulfoxide (DMSO), and a total of 1×106 cells/ml of H1975 cells were seeded in a 6-wells plate and incubated in RPMI-1640 medium containing Osimertinib. The initial concentrations of Osimertinib were started with a concentration equal to the half-maximal inhibitory concentration of H1975 cells. After a cycle of Osimertinib treatment, only a small percentage of cells remained. Once cells had resumed normal growth and returned to 80% confluence under the light microscope, the next cycle began. The drug concentration was gradually increased for the next cycle until cells could survive with 10 μM Osimertinib. After six months, the H1975OR cells were successfully established and were then harvested for RNAs analysis. **(B)** The images of gene probes detection were exhibited for illustration.

Supplementary Figure 4 Clinical significance of Notch signaling and Let-7b. Notch signaling factors of ADAM10, ADAM17, APH1A, APH1B, ARRDC1, CIR1, CTBP1, CTBP2, CUL1, DLL1, DLL3, DLL4, DTX1, DTX2, DTX3, DTX3L, DTX4, EP300, HDAC1, HDAC2, HES1, HES5, HEYL ITCH, JAG1, JAG2, KDM5A, LFNG, MAML1, MAML2, MAML3, MFNG, NCOR2, NCSTN, NOTCH1, NOTCH2, NOTCH3, NOTCH4, NRARP, NUMB, NUMBL, PSEN1, PSEN2, PSENEN, RBPJ, RBPJL, RFNG, SNW1, SPEN, HES2, HES4, HES7, HEY1, HEY2, were selected for analyzing in two studies of Lung Adenocarcinoma (TCGA, Firehose Legacy), and in Lung Adenocarcinoma (TCGA, Pan-Cancer Atlas). **(A)** Altered Notch signaling indicates shorter survival time, comparing to cases without Notch functional alternation. The clinical significance of Let-7b in patients with NSCLC was analyzed, and relative higher expression of Let-7b correlated with better survival expectance in lung adenocarcinoma **(B)**, but the indication was not significant in lung squamous carcinoma **(C)**.

Supplementary Figure 5 The source images of used blotting data in **Figure 1D**.

Supplementary Figure 6 The source images of used blotting data in **Figure 2E**. The grouping of gels/blots were cropped from different parts, and the full-length gels could be referred to in the **Supplemental Data**.

Supplementary Figure 7 The source images of used blotting data in **Figure 2F**. The grouping of gels/blots were cropped from different parts, and the full-length gels could be referred to in the **Supplemental Data**.

Supplementary Figure 8 The source images of used blotting data in **Figure 4C**. The grouping of gels/blots were cropped from different parts, and the full-length gels could be referred to in the **Supplemental Data**.

Supplementary Figure 9 The source images of used blotting data in **Figure 5F**. The grouping of gels/blots were cropped from different parts, and the full-length gels could be referred to in the **Supplemental Data**.

Supplementary Figure 10 The source images of used blotting data in **Figure 6C**. The grouping of gels/blots were cropped from different parts, and the full-length gels could be referred to in the **Supplemental Data**.
