# Correction: Stimulation of Let-7 maturation by metformin improved the response to tyrosine kinase inhibitor therapy in an m6A dependent manner

**DOI:** 10.3389/fonc.2026.1834833

**Published:** 2026-04-28

**Authors:** Kai Li, Shan Gao, Lei Ma, Ye Sun, Zi-Yang Peng, Jie Wu, Ning Du, Hong Ren, Shou-Ching Tang, Xin Sun

**Affiliations:** 1 Department of Thoracic Surgery, Department of Thoracic Surgery and Oncology, Cancer Center, The First Affiliated Hospital of Xi’an Jiaotong University, Xi’an City, China; 2 Department of Anesthesiology and Perioperative Medicine, Operating Centre, The First Affiliated Hospital of Xi’an Jiaotong University, Xi’an City, China; 3 Department of Anesthesiology and Operation, Operating Centre, The First Affiliated Hospital of Xi’an Jiaotong University, Xi’an City, China; 4 University of Mississippi Medical Center, Cancer Center and Research Institute, University of Mississippi, Jackson, MS, United States

**Keywords:** therapy resistance, cancer stem-like cells, tyrosine kinase inhibitor, n6-methyladenosine, miRNAs maturation

There was a mistake in **Figure 1** as published. In **Figures 1F**, the Western blot images contain incorrect band selections that do not accurately represent the original experimental results. The corrected **Figure 1** appears below.

**Figure 1:**
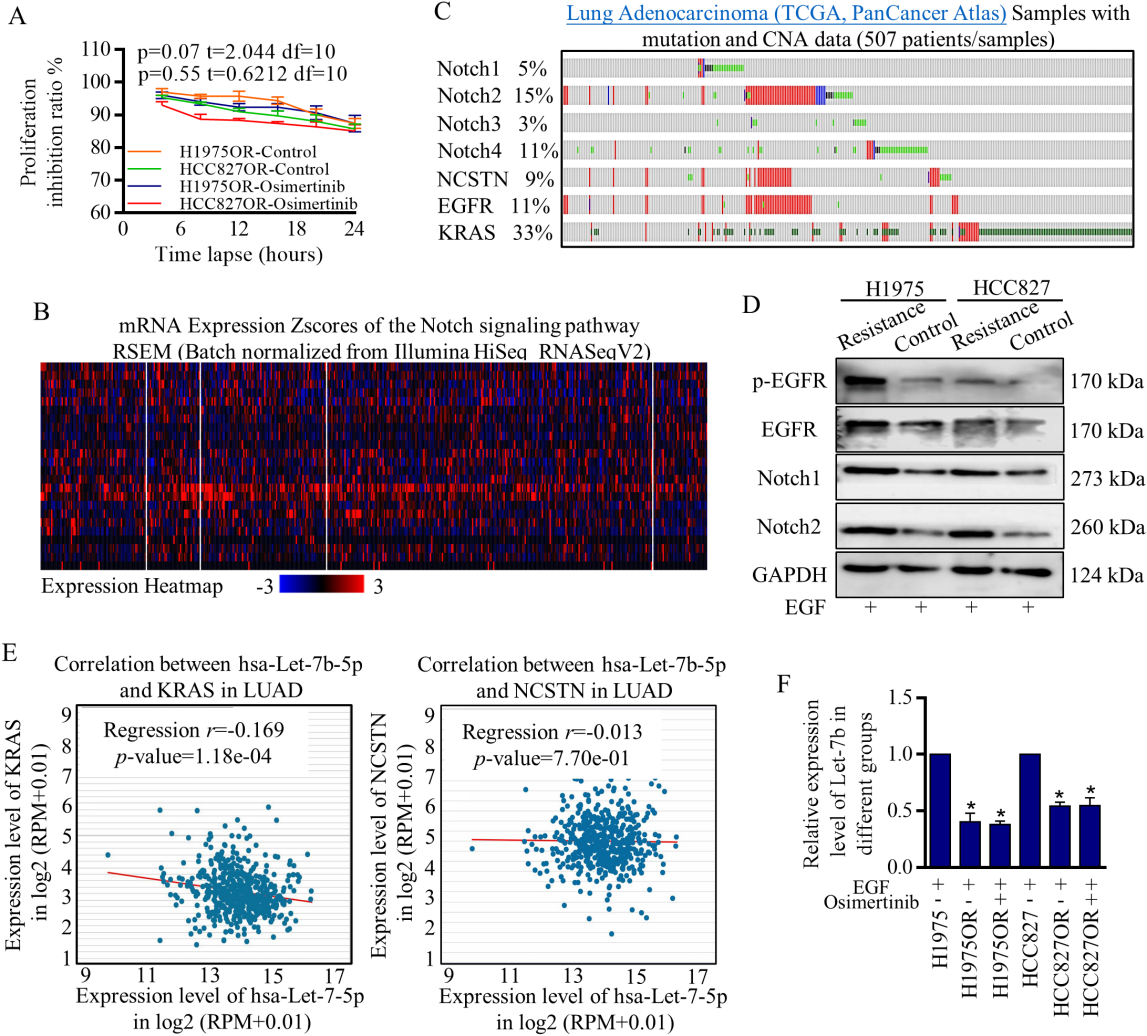
Notch signaling signatures and Let-7b expression deviations in Osimertinib resistant cells. **(A)** Proliferation inhibitive ratios were detected for defining the Osimertinib resistant H1975OR cells and HCC827OR cells. **(B)** The samples of lung Adenocarcinoma from the TCGA database (Pan-Cancer Atlas) were analyzed with mutation and CNA data, and Notch signaling participants were universally activated. **(C, D)** Key functional factors of Notch signaling were primarily screened and confirmed by western blotting, and Notch signaling factors were overexpressed in resistant cells (The grouping of gels/blots were cropped from different parts, and the full-length gels could be referred to in the supplemental data). **(E)** The inverse relationship between Let-7b and key Notch signaling activators was defining with using data from Star-Base Project. **(F)** Let-7b decreased significantly in Osimertinib resistant H1975OR and HCC827OR cells (**Figures 2F**).

There was a mistake in **Figure 1** as published. In **Figures 2E, F**, similar assembly errors were identified in these Western blot images. The corrected **Figure 2** appears below.

**Figure 2:**
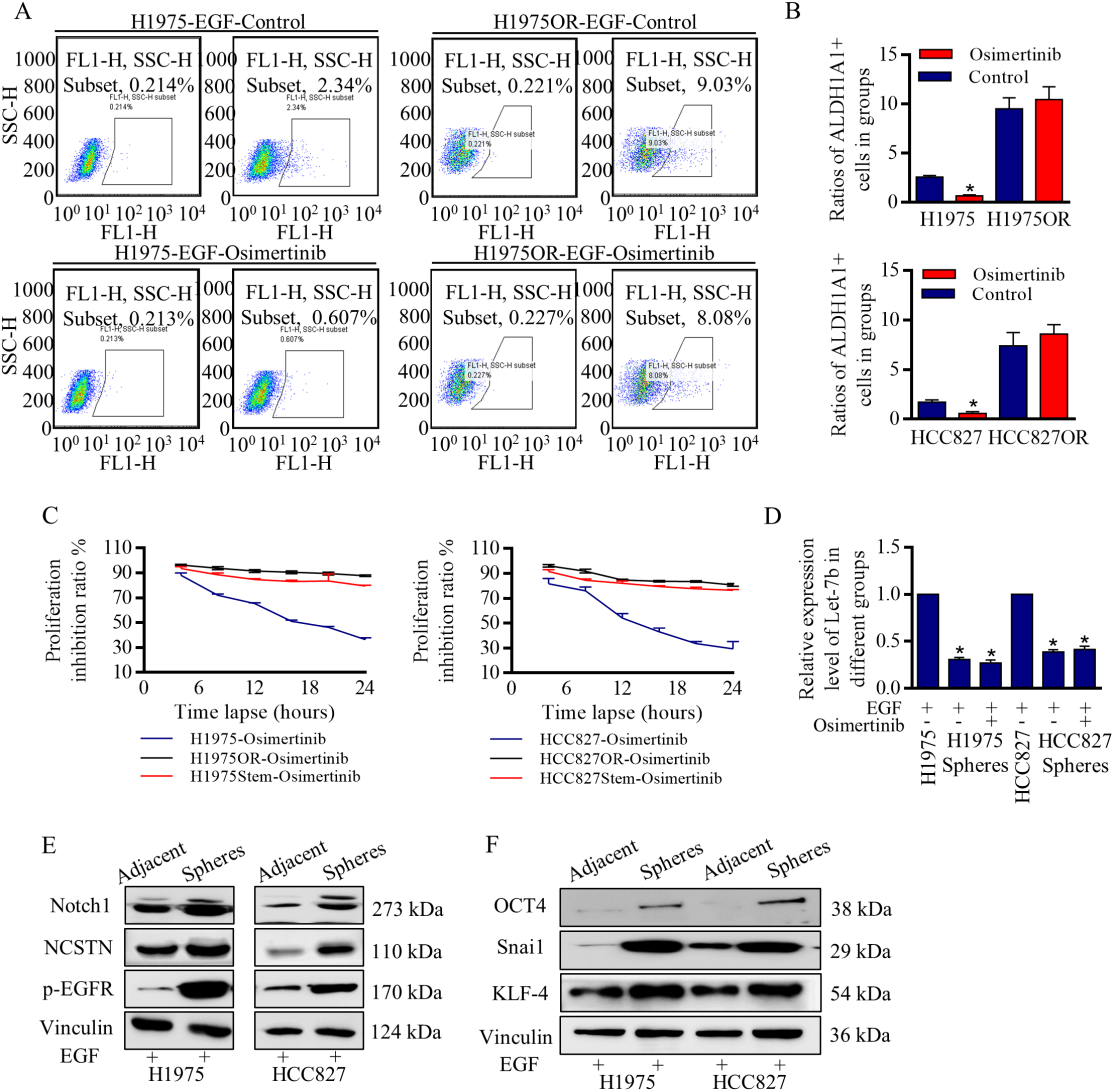
Notch signaling activation dependent stem cells renewal was responsible for Osimertinib resistance. **(A, B)** The ratio of ALDH1A1 (ALDH, ALDH1) positive cells accounted for less than 3% in H1975 and HCC827 cells, and Osimertinib decreased the ALDH1 positive stem cells ratio of H1975 and HCC827 effectively, however in H1975OR and HCC827OR cells, the ratios increased incredibly, and the stem cells ratio did not react to Osimertinib treatment. **(C)** Proliferation inhibition ratio was essentially the same between H1975 cells and H1975OR cells, between HCC827 cells and HCC827OR cells. **(D)** Let-7b decreased significantly in the spheres of H1975 cells and HCC827 cells, and Osimertinib did not change the Let-7b expression compared to the negative control. **(E, F)** Stem cells were identified with overexpressed stem associated markers, and the key notch signaling actors were excessively activated in spheres of stem cells (The grouping of gels/blots were cropped from different parts, and the full-length gels could be referred to in the Supplemental Data). *P < 0.05.

The Data Availability Statement was erroneously given as [The data that support the findings of this study are available from the corresponding author upon reasonable request. The established Osimertinib resistant adenocarcinoma cells of H1975OR were analyzed for gene-type signatures, which could be viewed at https://www.ncbi.nlm.nih.gov/geo/query/acc.cgi?acc=GSE184980.]. The correct Data Availability Statement is [The original contributions presented in this study are included in the article/supplementary material. The raw data supporting the conclusions of this article have been deposited in a public repository for verification. For the flow cytometry data, the original raw files (FCS files) are no longer available; however, the methodology and results are accurately presented in the manuscript. Further inquiries regarding raw data can be directed to the corresponding author, Dr. Xin Sun (dr_sun_endeavour@163.com), and will be assessed on a case-by-case basis following reasonable request.].

The original version of this article has been updated.

