# Supplementary figures and images for: Stimulation of Let-7 Maturation by Metformin Improved the Response to Tyrosine Kinase Inhibitor Therapy in an m6A Dependent Manner
Source: Front Oncol. 2022 Jan 6;11:731561. doi: 10.3389/fonc.2021.731561 (PMC8770959; doi:10.3389/fonc.2021.731561)

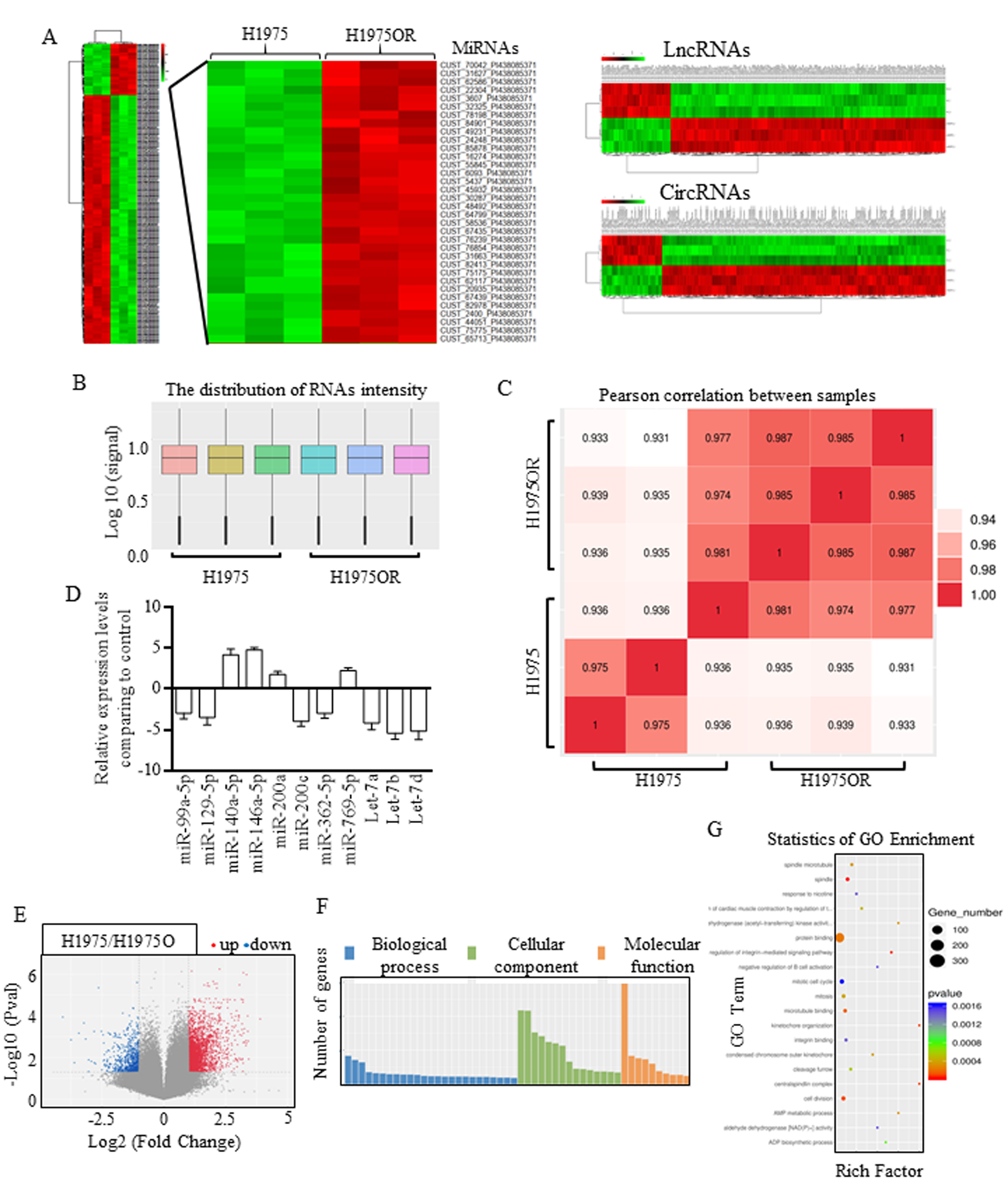

Supplement: Supplementary Figure 1 — RNA-panel analysis with gene ontology description and functional signaling predication. (A) More than 5000 non-coding RNAs were differentially expressed between the H1975 and H1975OR cells, and the representative heat map results were listed for illustrating the miRNAs, lncRNAs, circRNAs differences. (B) The distribution of RNA intensity in H1975 cells and H1975OR cells was confirmed. (C) Samples were detected triplicate for person correlation referring to quality control. (D) The abnormally expressed miRNAs were rechecked by qRT-PCR, and the expression variations of miRNAs were compared between qRT-PCR and microarray analysis results. (E) The volcano plot exhibited statistically significant differences in miRNAs expression. (F) Functional predication indicated the location and the pathways that alternative miRNAs may contribute to in the cellular biology and cancer initiation. (G) Signaling pathways that altered miRNAs may interact with were carried out with informatic GO analysis. [file Image_1.tif]

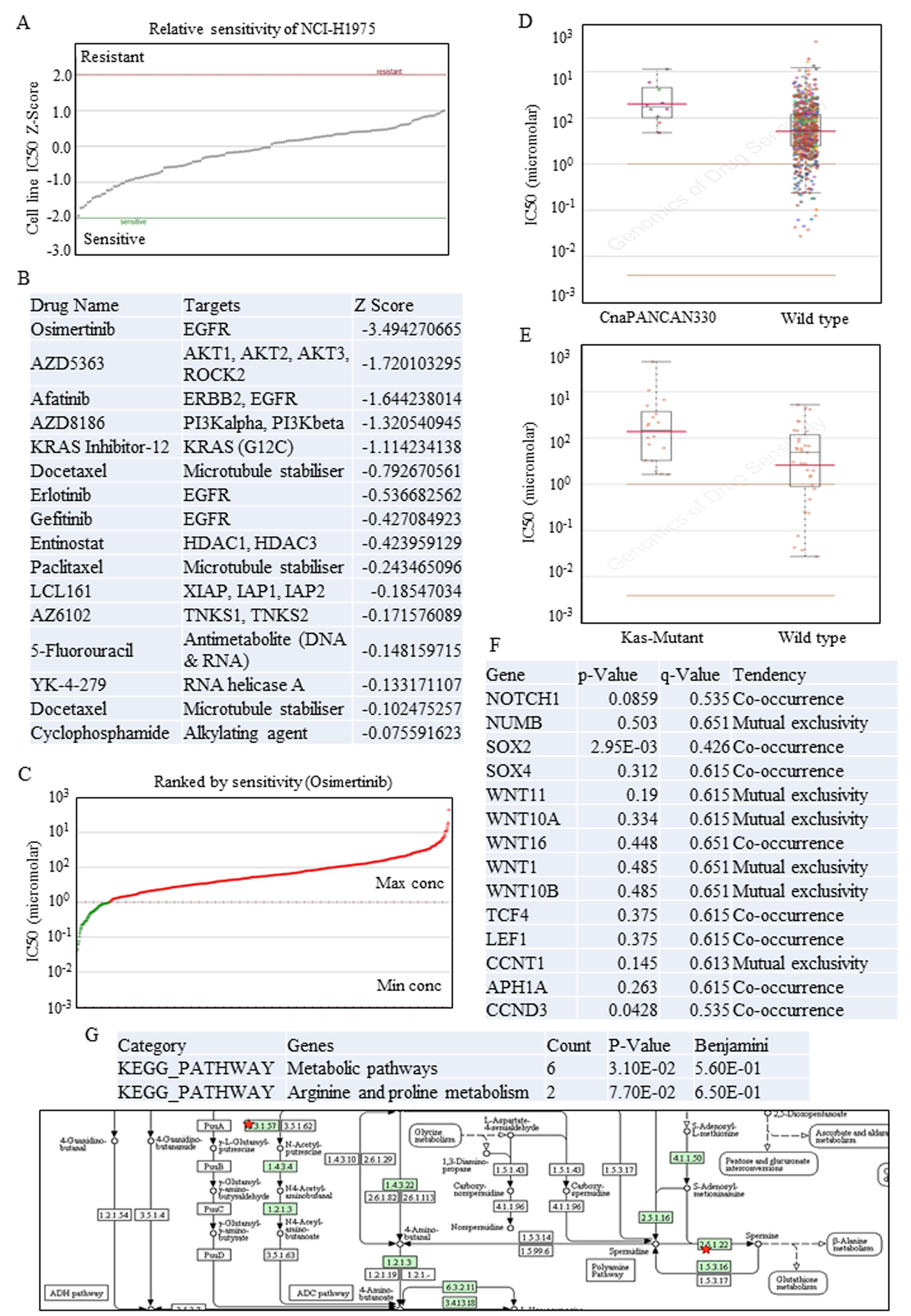

Supplement: Supplementary Figure 2 — The sensitivity of Osimertinib in treating Lung adenocarcinoma harboring mutant EGFR. The sensitivity analysis was carried out using shared data of Genomics of Drug Sensitivity in Cancer at the SANGER site. The Osimertinib sensitivity referring to lung cancer samples of PAN data was drafted with Z-score (A) and was rankly exhibited in table (B). (C) H1975 cells were sensitive to Osimertinib with concentration much lower than IC50. (D) EGFR mutant lung cancer cells are very sensitive to Osimertinib treatment, comparing to that of lung cancer cells with wild type EGFR. (E) Adenocarcinoma cells with Kas-Mutant status were resistant to Osimertinib, exhibiting much higher IC50 score. (F) Most of the Notch signaling associated factors were primarily identified with resistance. [file Image_2.tif]

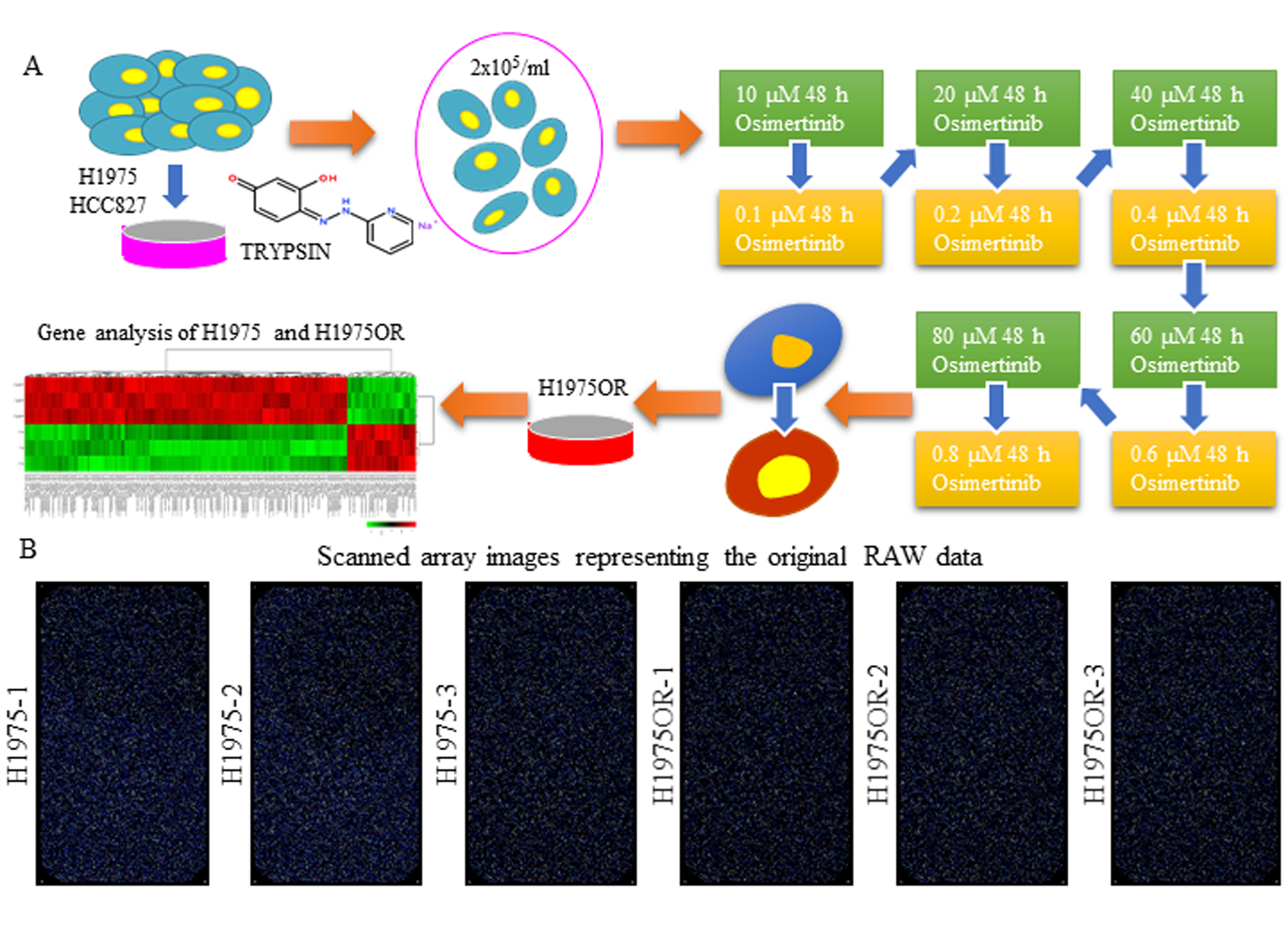

Supplement: Supplementary Figure 3 — Establishing the Osimertinib resistant cells. (A) Osimertinib was dissolved in dimethyl sulfoxide (DMSO), and a total of 1×10^6^ cells/ml of H1975 cells were seeded in a 6-wells plate and incubated in RPMI-1640 medium containing Osimertinib. The initial concentrations of Osimertinib were started with a concentration equal to the half-maximal inhibitory concentration of H1975 cells. After a cycle of Osimertinib treatment, only a small percentage of cells remained. Once cells had resumed normal growth and returned to 80% confluence under the light microscope, the next cycle began. The drug concentration was gradually increased for the next cycle until cells could survive with 10 μM Osimertinib. After six months, the H1975OR cells were successfully established and were then harvested for RNAs analysis. (B) The images of gene probes detection were exhibited for illustration. [file Image_3.tif]

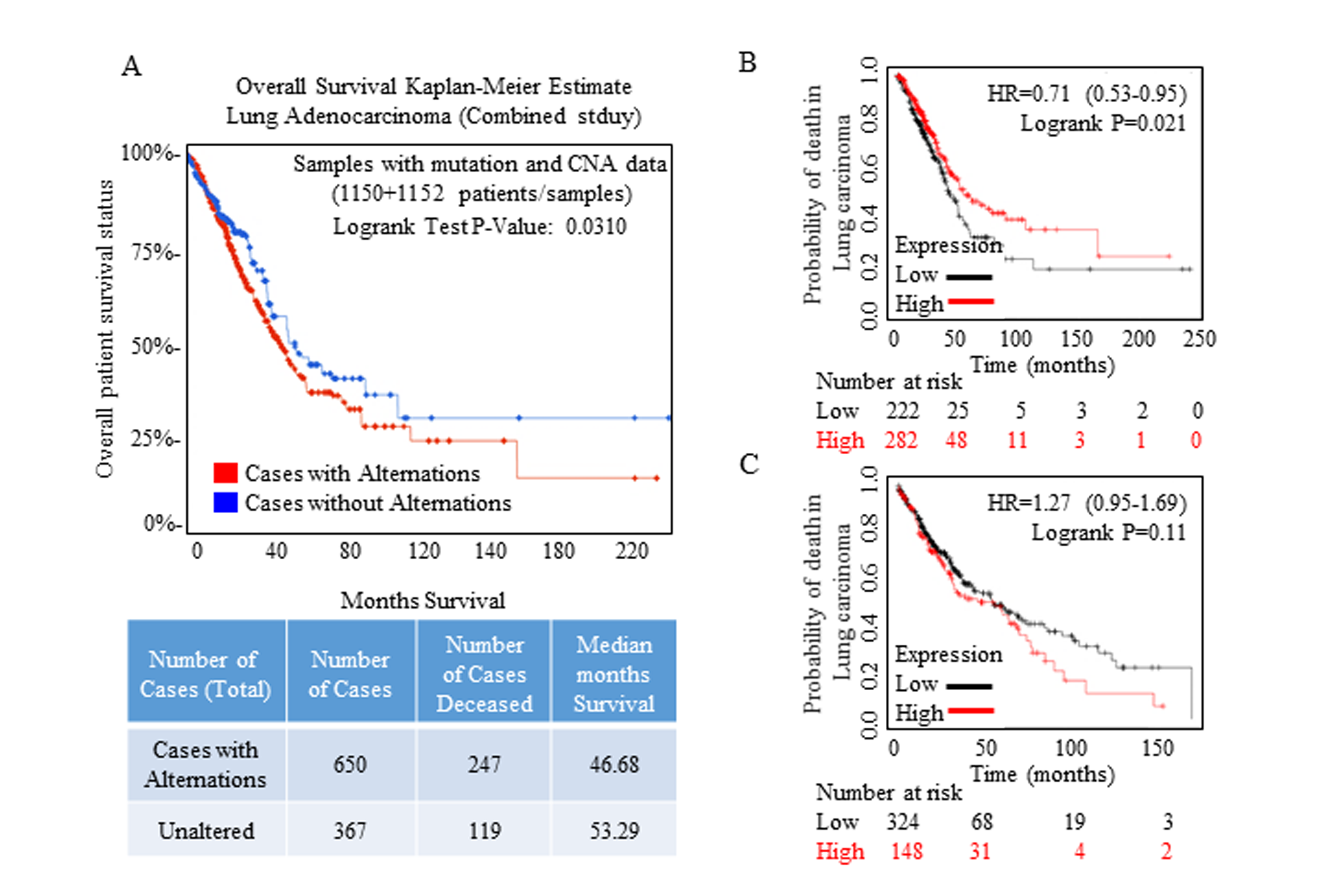

Supplement: Supplementary Figure 4 — Clinical significance of Notch signaling and Let-7b. Notch signaling factors of ADAM10, ADAM17, APH1A, APH1B, ARRDC1, CIR1, CTBP1, CTBP2, CUL1, DLL1, DLL3, DLL4, DTX1, DTX2, DTX3, DTX3L, DTX4, EP300, HDAC1, HDAC2, HES1, HES5, HEYL ITCH, JAG1, JAG2, KDM5A, LFNG, MAML1, MAML2, MAML3, MFNG, NCOR2, NCSTN, NOTCH1, NOTCH2, NOTCH3, NOTCH4, NRARP, NUMB, NUMBL, PSEN1, PSEN2, PSENEN, RBPJ, RBPJL, RFNG, SNW1, SPEN, HES2, HES4, HES7, HEY1, HEY2, were selected for analyzing in two studies of Lung Adenocarcinoma (TCGA, Firehose Legacy), and in Lung Adenocarcinoma (TCGA, Pan-Cancer Atlas). (A) Altered Notch signaling indicates shorter survival time, comparing to cases without Notch functional alternation. The clinical significance of Let-7b in patients with NSCLC was analyzed, and relative higher expression of Let-7b correlated with better survival expectance in lung adenocarcinoma (B), but the indication was not significant in lung squamous carcinoma (C). [file Image_4.tif]

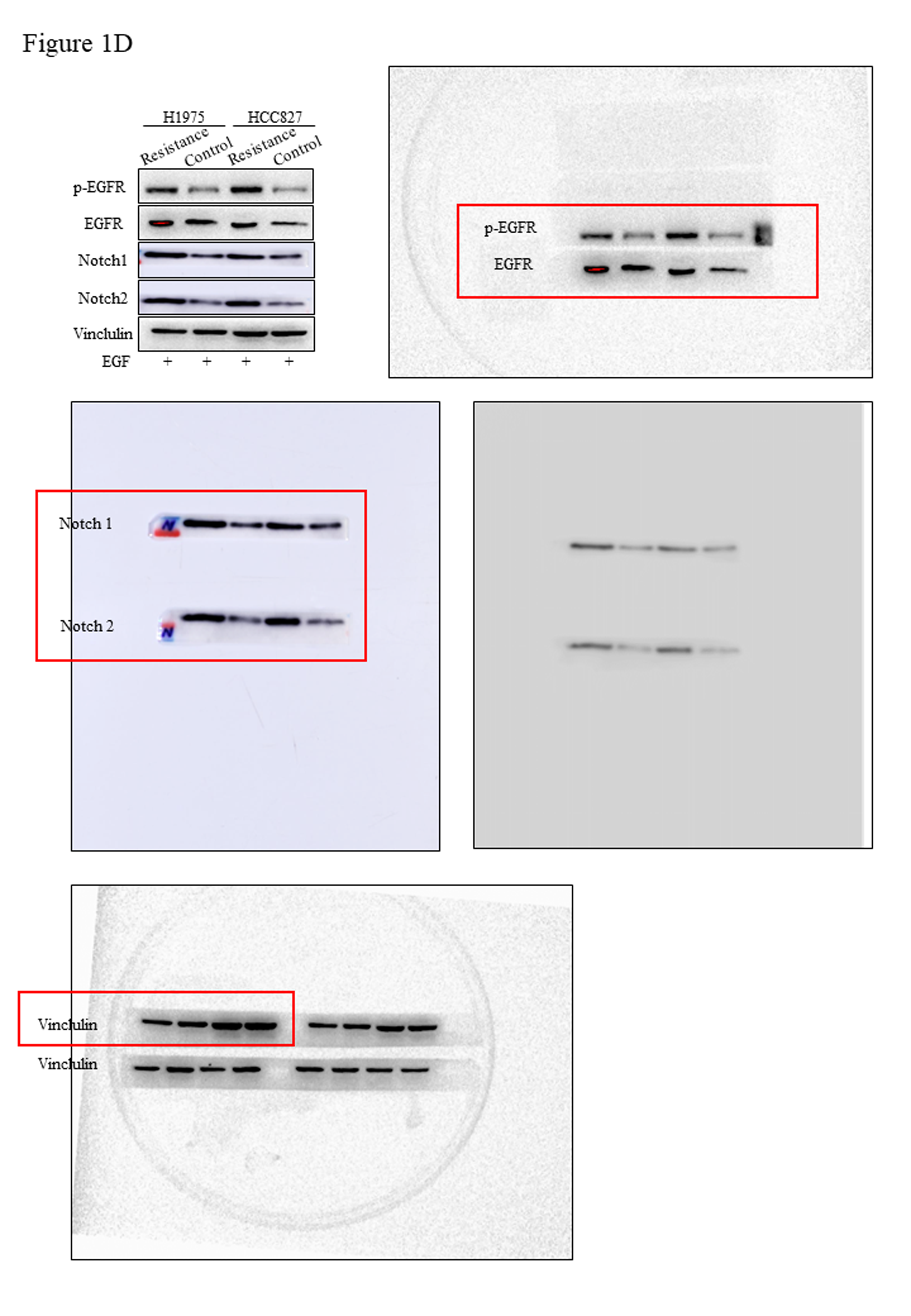

Supplement: Supplementary Figure 5 — The source images of used blotting data in Figure 1D . [file Image_5.tif]

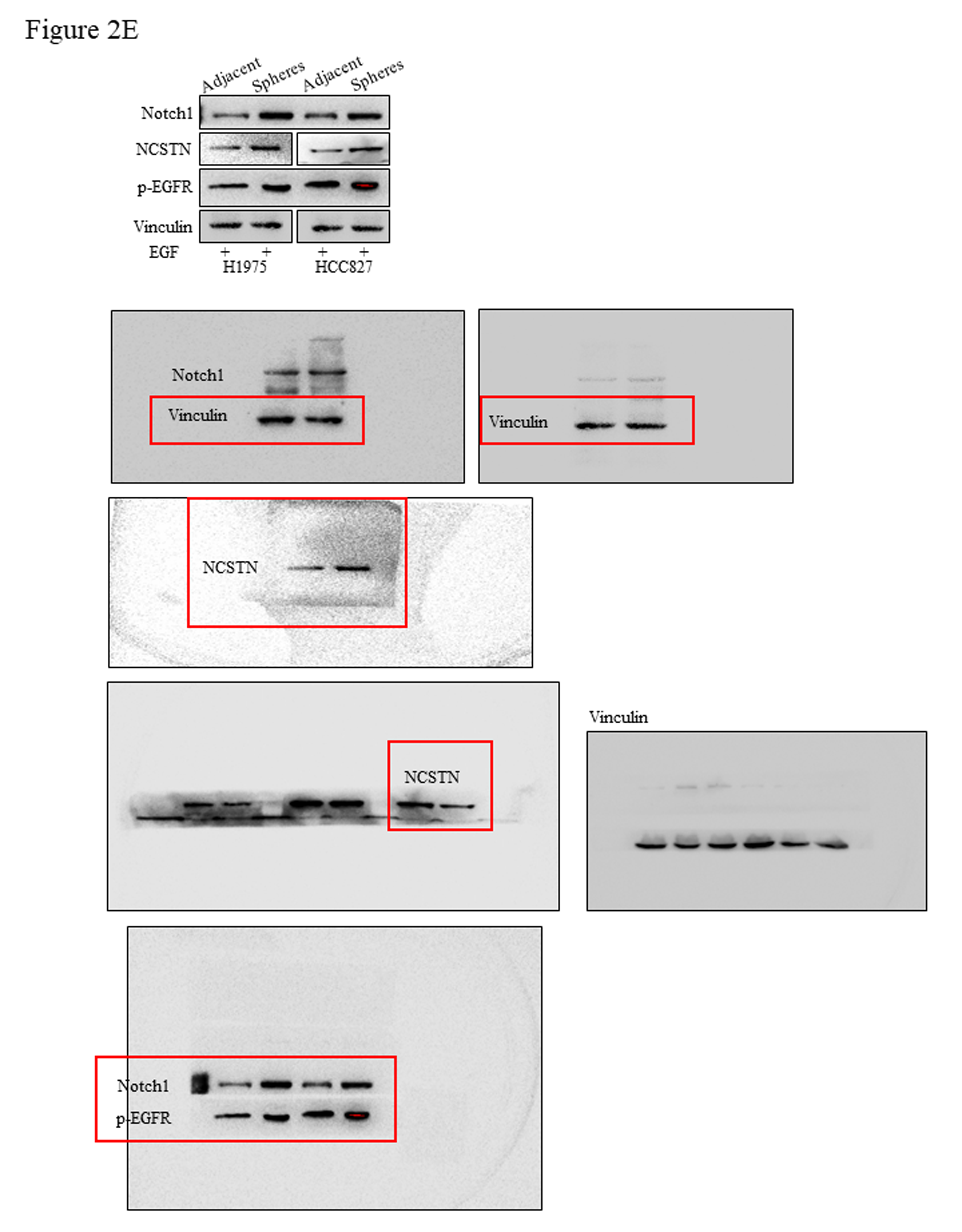

Supplement: Supplementary Figure 6 — The source images of used blotting data in Figure 2E . The grouping of gels/blots were cropped from different parts, and the full-length gels could be referred to in the Supplemental Data . [file Image_6.tif]

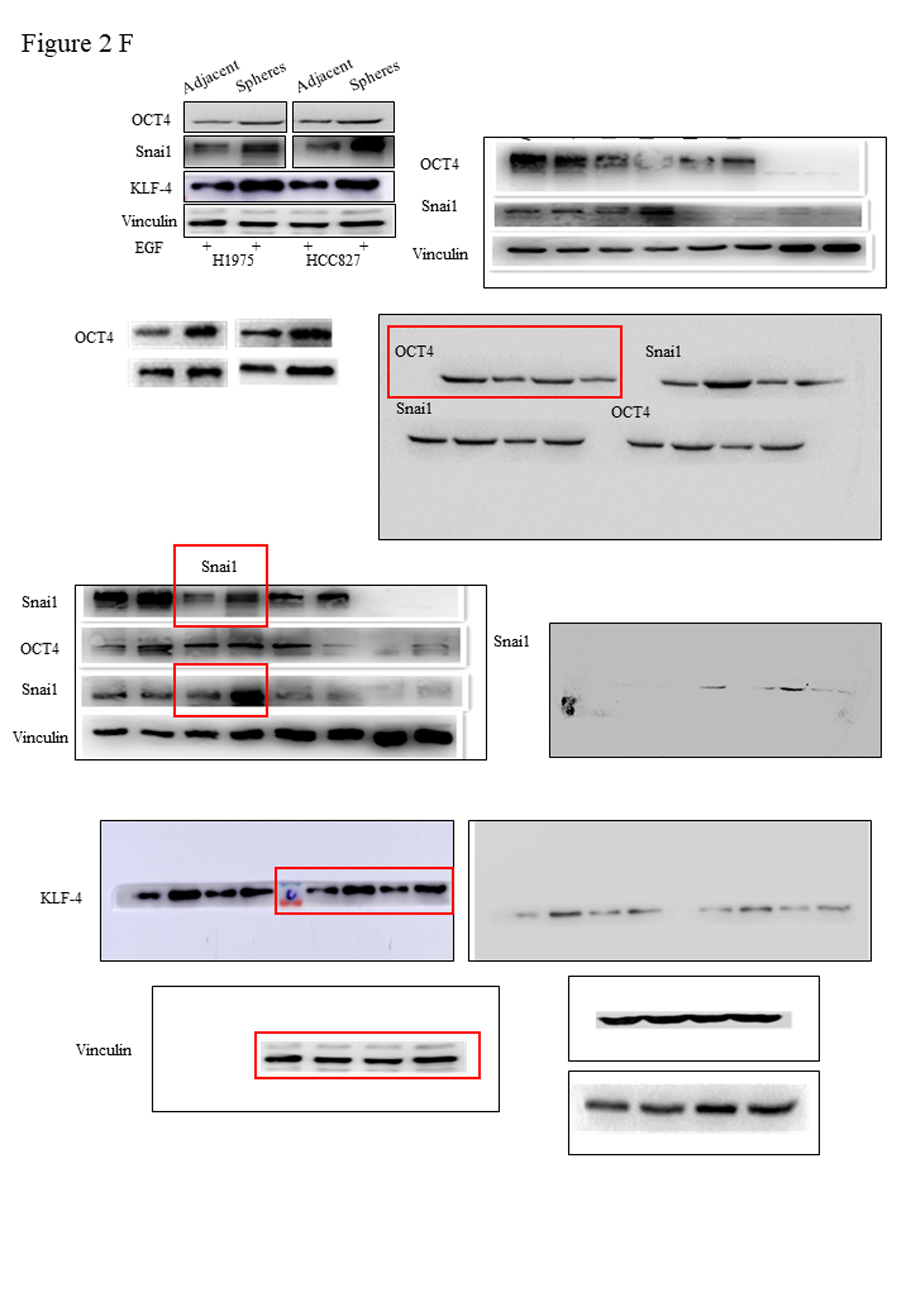

Supplement: Supplementary Figure 7 — The source images of used blotting data in Figure 2F . The grouping of gels/blots were cropped from different parts, and the full-length gels could be referred to in the Supplemental Data . [file Image_7.tif]

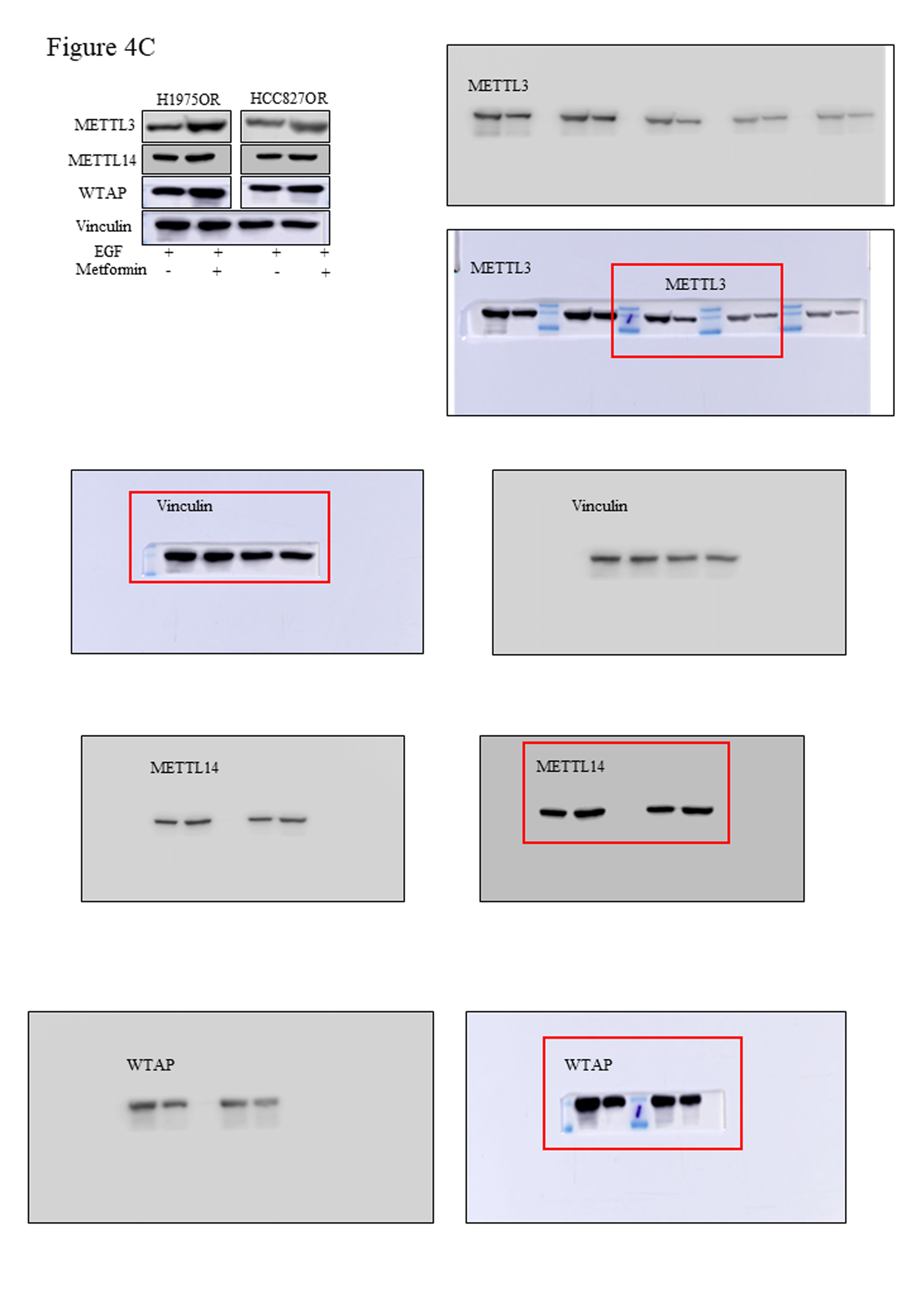

Supplement: Supplementary Figure 8 — The source images of used blotting data in Figure 4C . The grouping of gels/blots were cropped from different parts, and the full-length gels could be referred to in the Supplemental Data . [file Image_8.tif]

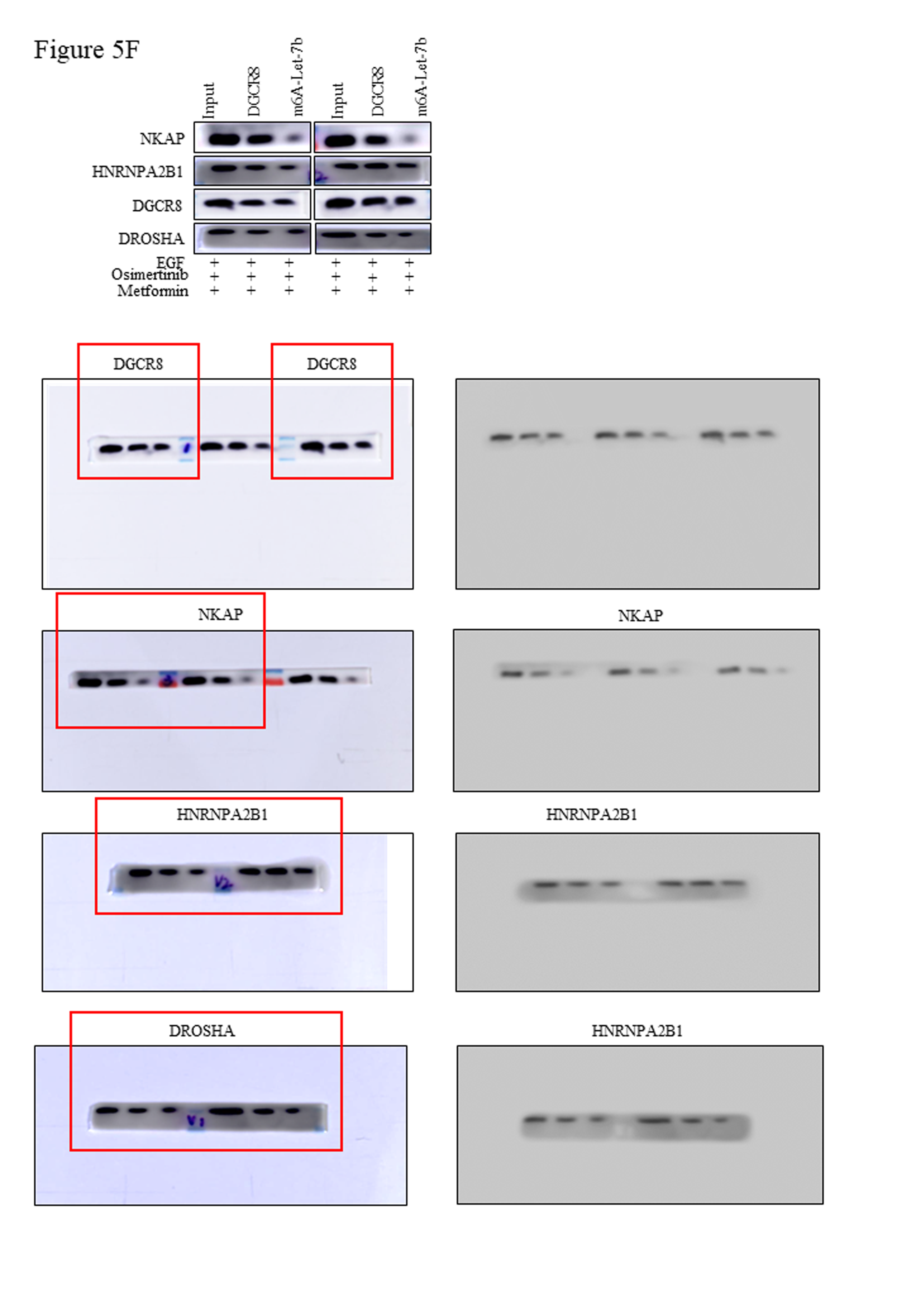

Supplement: Supplementary Figure 9 — The source images of used blotting data in Figure 5F . The grouping of gels/blots were cropped from different parts, and the full-length gels could be referred to in the Supplemental Data . [file Image_9.tif]

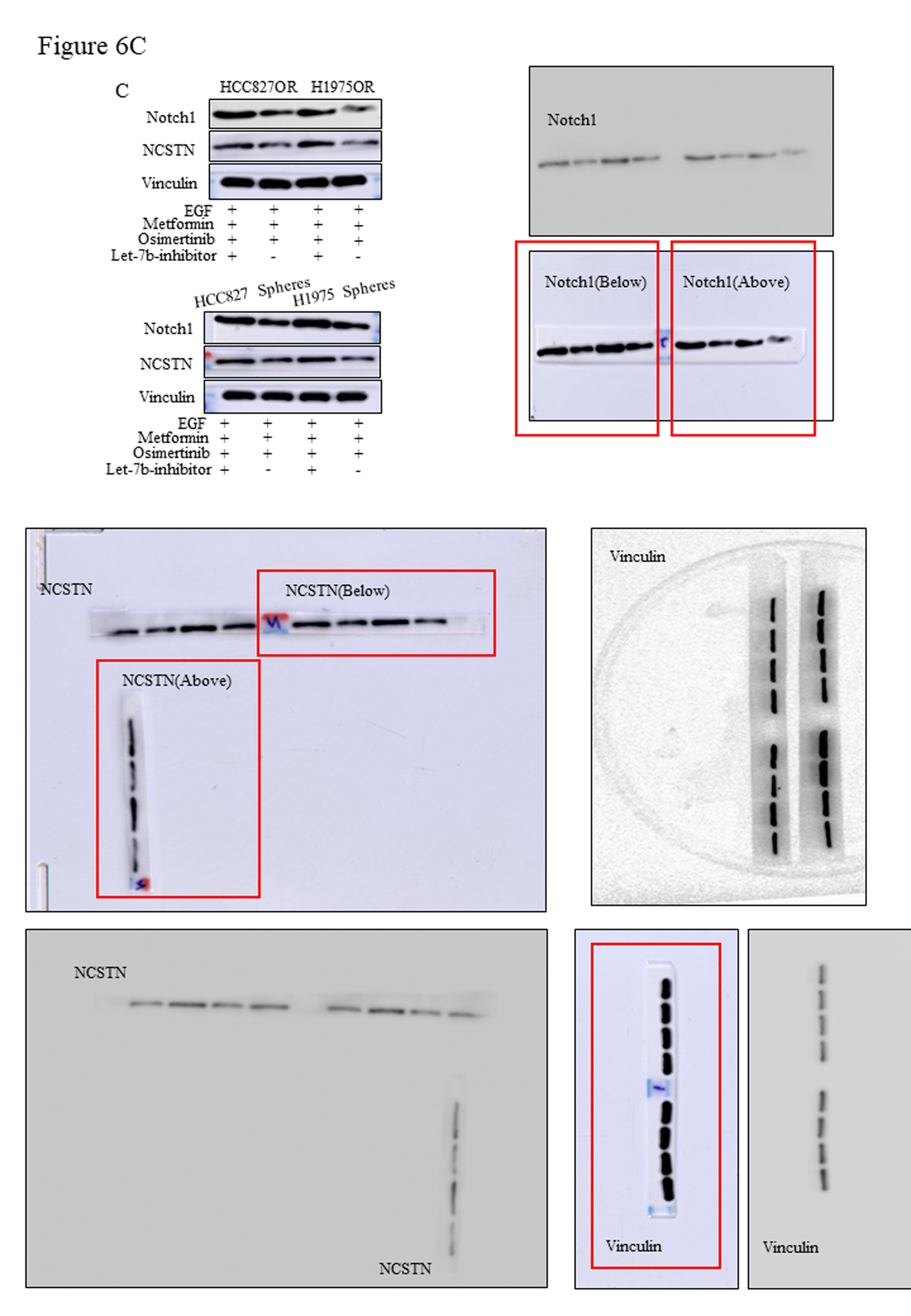

Supplement: Supplementary Figure 10 — The source images of used blotting data in Figure 6C . The grouping of gels/blots were cropped from different parts, and the full-length gels could be referred to in the Supplemental Data . [file Image_10.tif]
